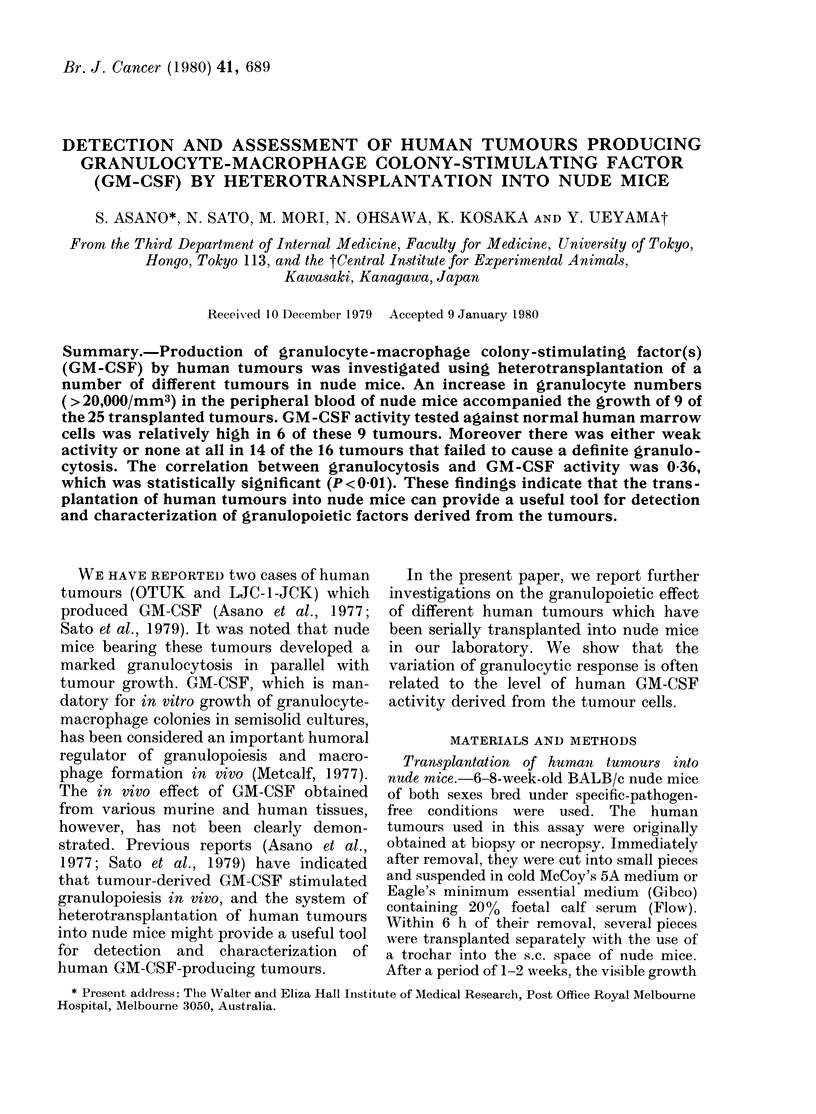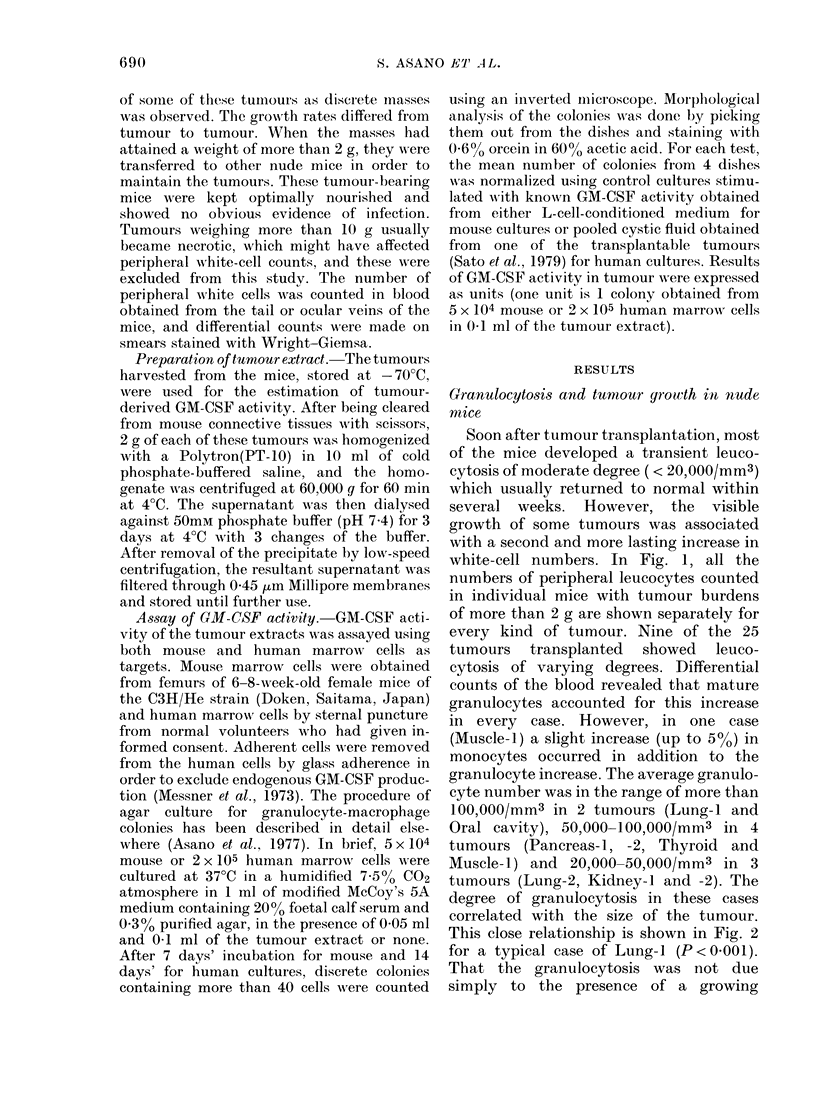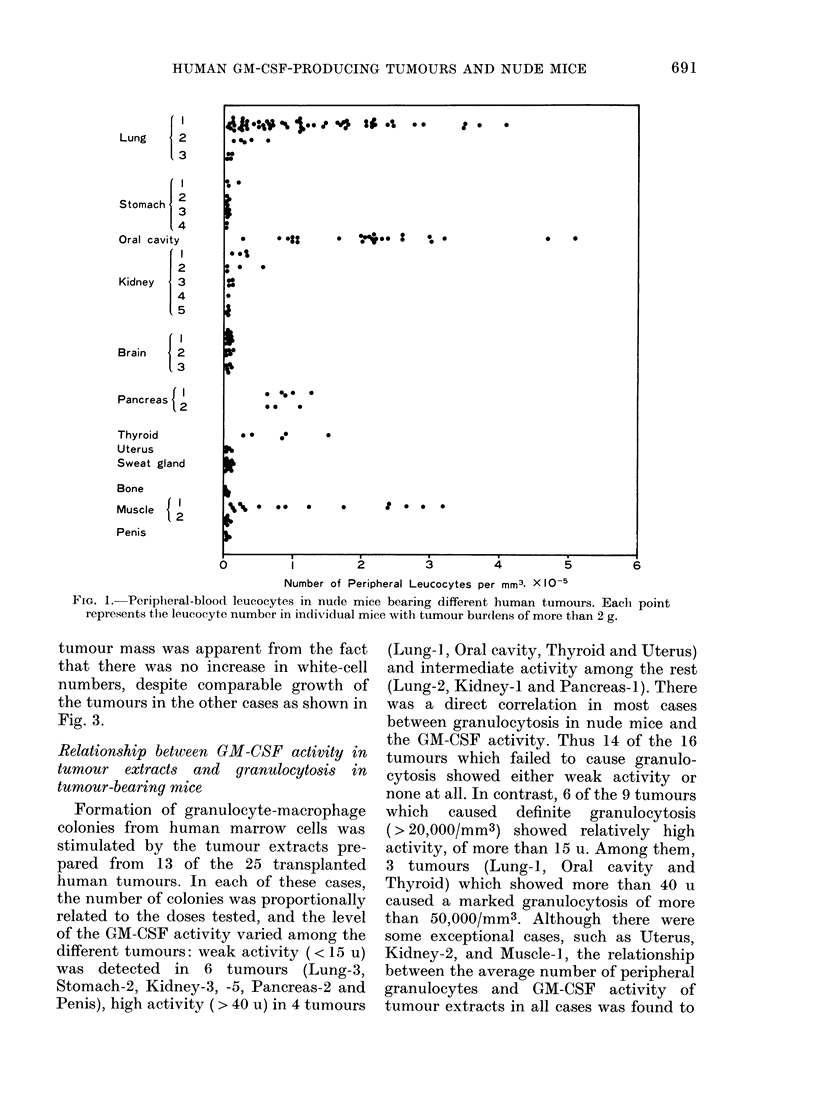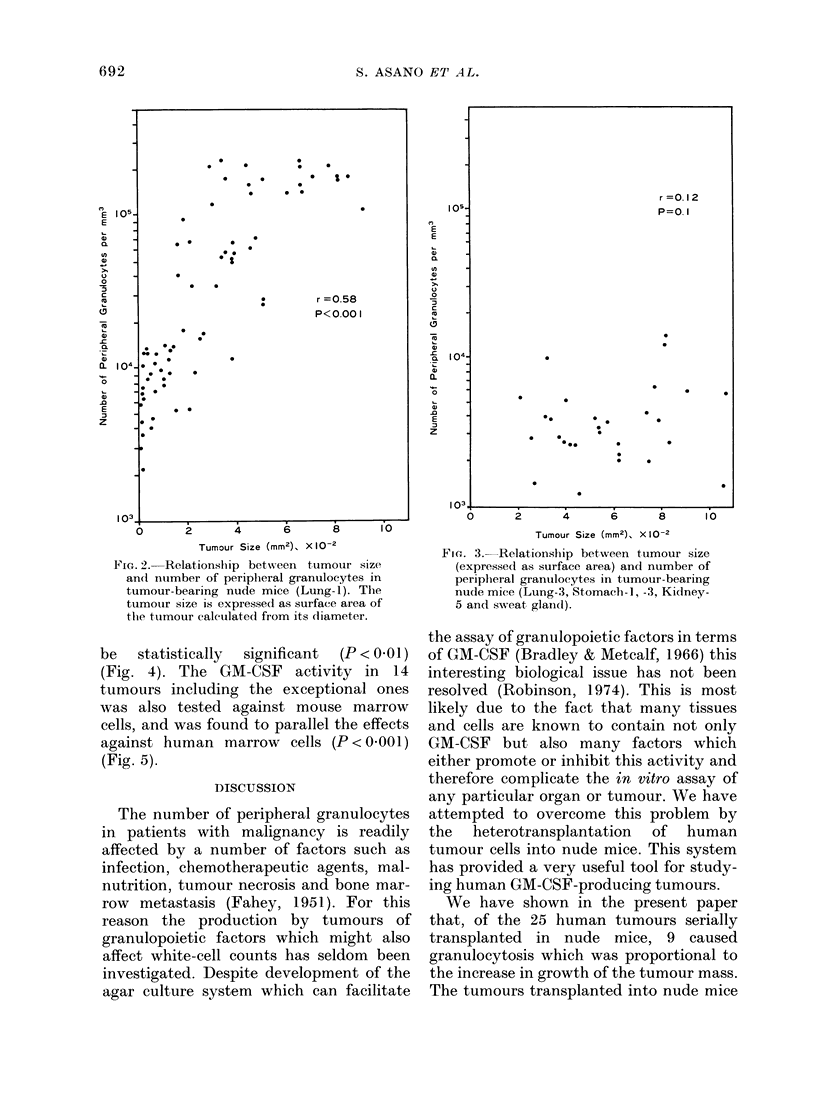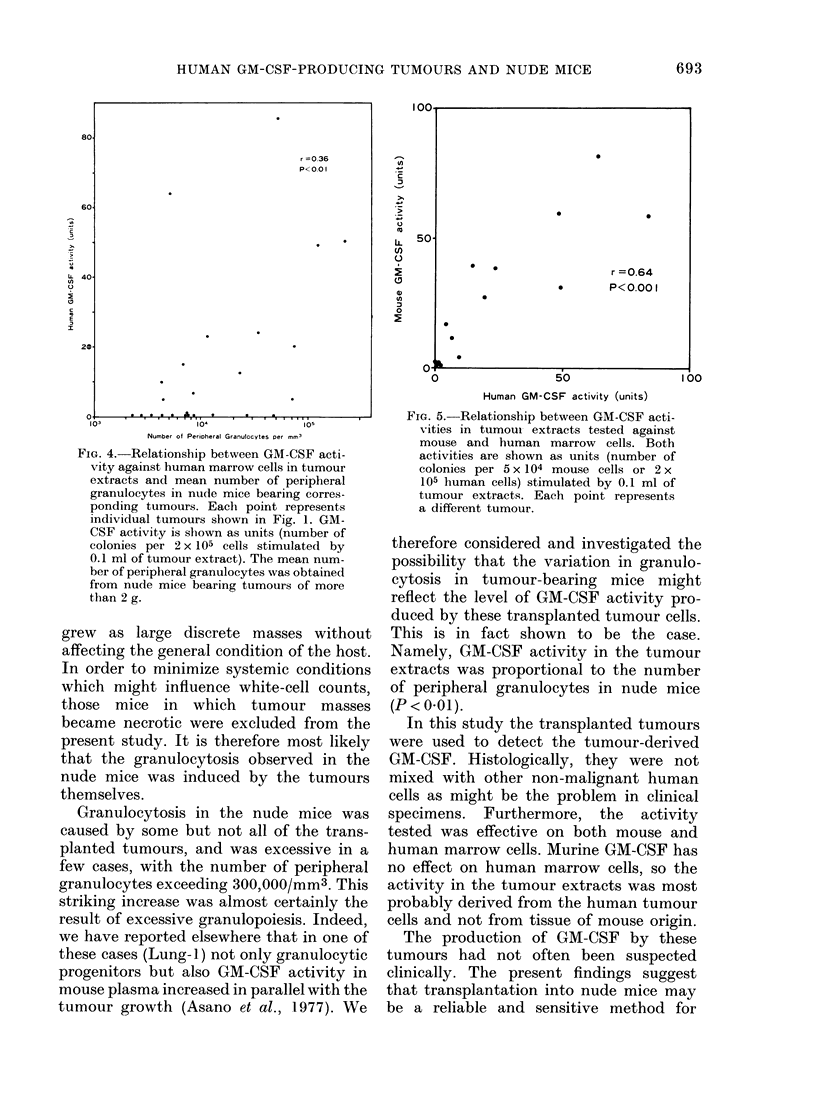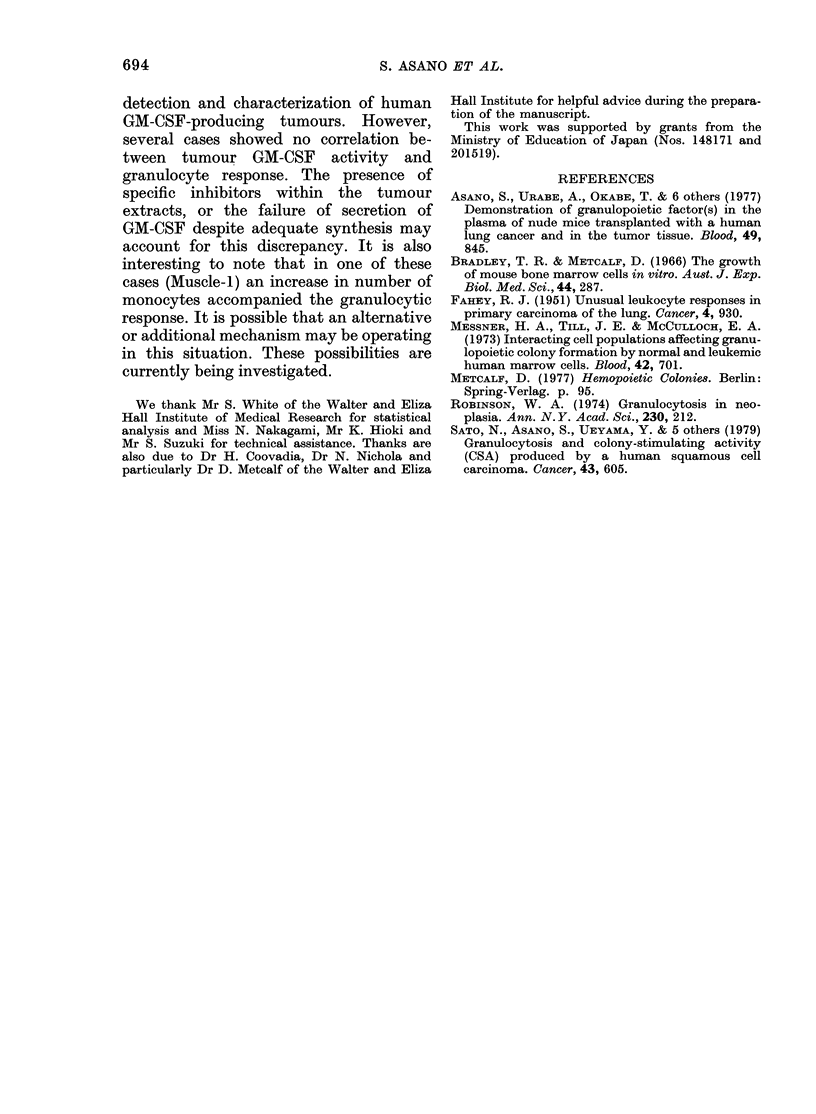# Detection and assessment of human tumours producing granulocyte-macrophage colony-stimulating factor (GM-CSF) by heterotransplantation into nude mice.

**DOI:** 10.1038/bjc.1980.130

**Published:** 1980-05

**Authors:** S. Asano, N. Sato, M. Mori, N. Ohsawa, K. Kosaka, Y. Ueyama

## Abstract

Production of granulocyte-macrophage colony-stimulating factor(s) (GM-CSF) by human tumours was investigated using heterotransplantation of a number of different tumours in nude mice. An increase in granulocyte numbers (> 20,000/mm3) in the peripheral blood of nude mice accompanied the growth of 9 of the 25 transplanted tumours. GM-CSF activity tested against normal human marrow cells was relatively high in 6 of these 9 tumours. Moreover there was either weak activity or none at all in 14 of the 16 tumours that failed to cause a definite granulocytosis. The correlation between granulocytosis and GM-CSF activity was 0.36, which was statistically significant (P < 0.01). These findings indicate that the transplantation of human tumours into nude mice can provide a useful tool for detection and characterization of granulopoietic factors derived from the tumours.


*Br. J. Cancer* (1980) **41**, 689

### DETECTION AND ASSESSMENT OF HUMAN TUMOURS PRODUCING GRANULOCYTE-MACROPHAGE COLONY-STIMULATING FACTOR (GM-CSF) BY HETEROTRANSPLANTATION INTO NUDE MICE

S. ASANO*, N. SATO, M. MORI, N. OHSAWA, K. KOSAKA AND Y. UEYAMA†

*From the Third Department of Internal Medicine, Faculty for Medicine, University of Tokyo, Hongo, Tokyo* 113, *and the* †*Central Institute for Experimental Animals, Kawasaki, Kanagawa, Japan*

Received 10 December 1979 Accepted 9 January 1980

**Summary.—Production of granulocyte-macrophage colony-stimulating factor(s) (GM-CSF) by human tumours was investigated using heterotransplantation of a number of different tumours in nude mice. An increase in granulocyte numbers (>20,000/mm³) in the peripheral blood of nude mice accompanied the growth of 9 of the 25 transplanted tumours. GM-CSF activity tested against normal human marrow cells was relatively high in 6 of these 9 tumours. Moreover there was either weak activity or none at all in 14 of the 16 tumours that failed to cause a definite granulocytosis. The correlation between granulocytosis and GM-CSF activity was 0·36, which was statistically significant ($P<0.01$). These findings indicate that the transplantation of human tumours into nude mice can provide a useful tool for detection and characterization of granulopoietic factors derived from the tumours.**

WE HAVE REPORTED two cases of human tumours (OTUK and LJC-1-JCK) which produced GM-CSF (Asano *et al.*, 1977; Sato *et al.*, 1979). It was noted that nude mice bearing these tumours developed a marked granulocytosis in parallel with tumour growth. GM-CSF, which is mandatory for *in vitro* growth of granulocyte-macrophage colonies in semisolid cultures, has been considered an important humoral regulator of granulopoiesis and macrophage formation *in vivo* (Metcalf, 1977). The *in vivo* effect of GM-CSF obtained from various murine and human tissues, however, has not been clearly demonstrated. Previous reports (Asano *et al.*, 1977; Sato *et al.*, 1979) have indicated that tumour-derived GM-CSF stimulated granulopoiesis *in vivo*, and the system of heterotransplantation of human tumours into nude mice might provide a useful tool for detection and characterization of human GM-CSF-producing tumours.

In the present paper, we report further investigations on the granulopoietic effect of different human tumours which have been serially transplanted into nude mice in our laboratory. We show that the variation of granulocytic response is often related to the level of human GM-CSF activity derived from the tumour cells.

### MATERIALS AND METHODS

*Transplantation of human tumours into nude mice.*—6–8-week-old BALB/c nude mice of both sexes bred under specific-pathogen-free conditions were used. The human tumours used in this assay were originally obtained at biopsy or necropsy. Immediately after removal, they were cut into small pieces and suspended in cold McCoy's 5A medium or Eagle's minimum essential medium (Gibco) containing 20% foetal calf serum (Flow). Within 6 h of their removal, several pieces were transplanted separately with the use of a trochar into the s.c. space of nude mice. After a period of 1–2 weeks, the visible growth

* Present address: The Walter and Eliza Hall Institute of Medical Research, Post Office Royal Melbourne Hospital, Melbourne 3050, Australia.

of some of these tumours as discrete masses was observed. The growth rates differed from tumour to tumour. When the masses had attained a weight of more than 2 g, they were transferred to other nude mice in order to maintain the tumours. These tumour-bearing mice were kept optimally nourished and showed no obvious evidence of infection. Tumours weighing more than 10 g usually became necrotic, which might have affected peripheral white-cell counts, and these were excluded from this study. The number of peripheral white cells was counted in blood obtained from the tail or ocular veins of the mice, and differential counts were made on smears stained with Wright–Giemsa.

*Preparation of tumour extract.*—The tumours harvested from the mice, stored at −70°C, were used for the estimation of tumour-derived GM-CSF activity. After being cleared from mouse connective tissues with scissors, 2 g of each of these tumours was homogenized with a Polytron(PT-10) in 10 ml of cold phosphate-buffered saline, and the homogenate was centrifuged at 60,000 *g* for 60 min at 4°C. The supernatant was then dialysed against 50mM phosphate buffer (pH 7·4) for 3 days at 4°C with 3 changes of the buffer. After removal of the precipitate by low-speed centrifugation, the resultant supernatant was filtered through 0·45 μm Millipore membranes and stored until further use.

*Assay of GM-CSF activity.*—GM-CSF activity of the tumour extracts was assayed using both mouse and human marrow cells as targets. Mouse marrow cells were obtained from femurs of 6–8-week-old female mice of the C3H/He strain (Doken, Saitama, Japan) and human marrow cells by sternal puncture from normal volunteers who had given informed consent. Adherent cells were removed from the human cells by glass adherence in order to exclude endogenous GM-CSF production (Messner *et al.*, 1973). The procedure of agar culture for granulocyte-macrophage colonies has been described in detail elsewhere (Asano *et al.*, 1977). In brief, $5 \times 10^4$ mouse or $2 \times 10^5$ human marrow cells were cultured at 37°C in a humidified 7·5% $CO_2$ atmosphere in 1 ml of modified McCoy's 5A medium containing 20% foetal calf serum and 0·3% purified agar, in the presence of 0·05 ml and 0·1 ml of the tumour extract or none. After 7 days' incubation for mouse and 14 days' for human cultures, discrete colonies containing more than 40 cells were counted using an inverted microscope. Morphological analysis of the colonies was done by picking them out from the dishes and staining with 0·6% orcein in 60% acetic acid. For each test, the mean number of colonies from 4 dishes was normalized using control cultures stimulated with known GM-CSF activity obtained from either L-cell-conditioned medium for mouse cultures or pooled cystic fluid obtained from one of the transplantable tumours (Sato *et al.*, 1979) for human cultures. Results of GM-CSF activity in tumour were expressed as units (one unit is 1 colony obtained from $5 \times 10^4$ mouse or $2 \times 10^5$ human marrow cells in 0·1 ml of the tumour extract).

### RESULTS

### *Granulocytosis and tumour growth in nude mice*

Soon after tumour transplantation, most of the mice developed a transient leucocytosis of moderate degree ($< 20,000/mm^3$) which usually returned to normal within several weeks. However, the visible growth of some tumours was associated with a second and more lasting increase in white-cell numbers. In Fig. 1, all the numbers of peripheral leucocytes counted in individual mice with tumour burdens of more than 2 g are shown separately for every kind of tumour. Nine of the 25 tumours transplanted showed leucocytosis of varying degrees. Differential counts of the blood revealed that mature granulocytes accounted for this increase in every case. However, in one case (Muscle-1) a slight increase (up to 5%) in monocytes occurred in addition to the granulocyte increase. The average granulocyte number was in the range of more than $100,000/mm^3$ in 2 tumours (Lung-1 and Oral cavity), $50,000–100,000/mm^3$ in 4 tumours (Pancreas-1, -2, Thyroid and Muscle-1) and $20,000–50,000/mm^3$ in 3 tumours (Lung-2, Kidney-1 and -2). The degree of granulocytosis in these cases correlated with the size of the tumour. This close relationship is shown in Fig. 2 for a typical case of Lung-1 ($P < 0·001$). That the granulocytosis was not due simply to the presence of a growing

FIG. 1.—Peripheral-blood leucocytes in nude mice bearing different human tumours. Each point represents the leucocyte number in individual mice with tumour burdens of more than 2 g.

tumour mass was apparent from the fact that there was no increase in white-cell numbers, despite comparable growth of the tumours in the other cases as shown in Fig. 3.

*Relationship between GM-CSF activity in tumour extracts and granulocytosis in tumour-bearing mice*

Formation of granulocyte-macrophage colonies from human marrow cells was stimulated by the tumour extracts prepared from 13 of the 25 transplanted human tumours. In each of these cases, the number of colonies was proportionally related to the doses tested, and the level of the GM-CSF activity varied among the different tumours: weak activity (< 15 u) was detected in 6 tumours (Lung-3, Stomach-2, Kidney-3, -5, Pancreas-2 and Penis), high activity (> 40 u) in 4 tumours (Lung-1, Oral cavity, Thyroid and Uterus) and intermediate activity among the rest (Lung-2, Kidney-1 and Pancreas-1). There was a direct correlation in most cases between granulocytosis in nude mice and the GM-CSF activity. Thus 14 of the 16 tumours which failed to cause granulocytosis showed either weak activity or none at all. In contrast, 6 of the 9 tumours which caused definite granulocytosis (> 20,000/mm$^3$) showed relatively high activity, of more than 15 u. Among them, 3 tumours (Lung-1, Oral cavity and Thyroid) which showed more than 40 u caused a marked granulocytosis of more than 50,000/mm$^3$. Although there were some exceptional cases, such as Uterus, Kidney-2, and Muscle-1, the relationship between the average number of peripheral granulocytes and GM-CSF activity of tumour extracts in all cases was found to

FIG. 2.—Relationship between tumour size and number of peripheral granulocytes in tumour-bearing nude mice (Lung-1). The tumour size is expressed as surface area of the tumour calculated from its diameter.

FIG. 3.—Relationship between tumour size (expressed as surface area) and number of peripheral granulocytes in tumour-bearing nude mice (Lung-3, Stomach-1, -3, Kidney-5 and sweat gland).

be statistically significant ($P < 0{\cdot}01$) (Fig. 4). The GM-CSF activity in 14 tumours including the exceptional ones was also tested against mouse marrow cells, and was found to parallel the effects against human marrow cells ($P < 0{\cdot}001$) (Fig. 5).

### DISCUSSION

The number of peripheral granulocytes in patients with malignancy is readily affected by a number of factors such as infection, chemotherapeutic agents, malnutrition, tumour necrosis and bone marrow metastasis (Fahey, 1951). For this reason the production by tumours of granulopoietic factors which might also affect white-cell counts has seldom been investigated. Despite development of the agar culture system which can facilitate the assay of granulopoietic factors in terms of GM-CSF (Bradley & Metcalf, 1966) this interesting biological issue has not been resolved (Robinson, 1974). This is most likely due to the fact that many tissues and cells are known to contain not only GM-CSF but also many factors which either promote or inhibit this activity and therefore complicate the *in vitro* assay of any particular organ or tumour. We have attempted to overcome this problem by the heterotransplantation of human tumour cells into nude mice. This system has provided a very useful tool for studying human GM-CSF-producing tumours.

We have shown in the present paper that, of the 25 human tumours serially transplanted in nude mice, 9 caused granulocytosis which was proportional to the increase in growth of the tumour mass. The tumours transplanted into nude mice

FIG. 4.—Relationship between GM-CSF activity against human marrow cells in tumour extracts and mean number of peripheral granulocytes in nude mice bearing corresponding tumours. Each point represents individual tumours shown in Fig. 1. GM-CSF activity is shown as units (number of colonies per $2 \times 10^5$ cells stimulated by 0.1 ml of tumour extract). The mean number of peripheral granulocytes was obtained from nude mice bearing tumours of more than 2 g.

FIG. 5.—Relationship between GM-CSF activities in tumour extracts tested against mouse and human marrow cells. Both activities are shown as units (number of colonies per $5 \times 10^4$ mouse cells or $2 \times 10^5$ human cells) stimulated by 0.1 ml of tumour extracts. Each point represents a different tumour.

grew as large discrete masses without affecting the general condition of the host. In order to minimize systemic conditions which might influence white-cell counts, those mice in which tumour masses became necrotic were excluded from the present study. It is therefore most likely that the granulocytosis observed in the nude mice was induced by the tumours themselves.

Granulocytosis in the nude mice was caused by some but not all of the transplanted tumours, and was excessive in a few cases, with the number of peripheral granulocytes exceeding 300,000/mm³. This striking increase was almost certainly the result of excessive granulopoiesis. Indeed, we have reported elsewhere that in one of these cases (Lung-1) not only granulocytic progenitors but also GM-CSF activity in mouse plasma increased in parallel with the tumour growth (Asano *et al.*, 1977). We therefore considered and investigated the possibility that the variation in granulocytosis in tumour-bearing mice might reflect the level of GM-CSF activity produced by these transplanted tumour cells. This is in fact shown to be the case. Namely, GM-CSF activity in the tumour extracts was proportional to the number of peripheral granulocytes in nude mice ($P < 0{\cdot}01$).

In this study the transplanted tumours were used to detect the tumour-derived GM-CSF. Histologically, they were not mixed with other non-malignant human cells as might be the problem in clinical specimens. Furthermore, the activity tested was effective on both mouse and human marrow cells. Murine GM-CSF has no effect on human marrow cells, so the activity in the tumour extracts was most probably derived from the human tumour cells and not from tissue of mouse origin.

The production of GM-CSF by these tumours had not often been suspected clinically. The present findings suggest that transplantation into nude mice may be a reliable and sensitive method for detection and characterization of human GM-CSF-producing tumours. However, several cases showed no correlation between tumour GM-CSF activity and granulocyte response. The presence of specific inhibitors within the tumour extracts, or the failure of secretion of GM-CSF despite adequate synthesis may account for this discrepancy. It is also interesting to note that in one of these cases (Muscle-1) an increase in number of monocytes accompanied the granulocytic response. It is possible that an alternative or additional mechanism may be operating in this situation. These possibilities are currently being investigated.

We thank Mr S. White of the Walter and Eliza Hall Institute of Medical Research for statistical analysis and Miss N. Nakagami, Mr K. Hioki and Mr S. Suzuki for technical assistance. Thanks are also due to Dr H. Coovadia, Dr N. Nichola and particularly Dr D. Metcalf of the Walter and Eliza Hall Institute for helpful advice during the preparation of the manuscript.

This work was supported by grants from the Ministry of Education of Japan (Nos. 148171 and 201519).

### REFERENCES

Asano, S., Urabe, A., Okabe, T. & 6 others (1977) Demonstration of granulopoietic factor(s) in the plasma of nude mice transplanted with a human lung cancer and in the tumor tissue. *Blood*, **49**, 845.

Bradley, T. R. & Metcalf, D. (1966) The growth of mouse bone marrow cells *in vitro*. *Aust. J. Exp. Biol. Med. Sci.*, **44**, 287.

Fahey, R. J. (1951) Unusual leukocyte responses in primary carcinoma of the lung. *Cancer*, **4**, 930.

Messner, H. A., Till, J. E. & McCulloch, E. A. (1973) Interacting cell populations affecting granulopoietic colony formation by normal and leukemic human marrow cells. *Blood*, **42**, 701.

Metcalf, D. (1977) *Hemopoietic Colonies*. Berlin: Spring-Verlag. p. 95.

Robinson, W. A. (1974) Granulocytosis in neoplasia. *Ann. N.Y. Acad. Sci.*, **230**, 212.

Sato, N., Asano, S., Ueyama, Y. & 5 others (1979) Granulocytosis and colony-stimulating activity (CSA) produced by a human squamous cell carcinoma. *Cancer*, **43**, 605.